# Personalized Kirigami Strain Sensors for in vivo Applications

**DOI:** 10.1002/aisy.202400886

**Published:** 2025-02-23

**Authors:** Siheng Sean You, Max Schober, Soufyane Ben-Ayed, Sahab Babaee, Stephanie Owyang, Josh Jenkins, Peter R. Chai, Giovanni Traverso

**Affiliations:** David H. Koch Institute for Integrative Cancer Research, Massachusetts Institute of Technology, Cambridge, MA 02139, USA; Division of Gastroenterology, Hepatology and Endoscopy, Brigham and Women’s Hospital, Harvard Medical School, Boston, MA 02115, USA; Division of Gastroenterology, Hepatology and Endoscopy, Brigham and Women’s Hospital, Harvard Medical School, Boston, MA 02115, USA; The Department of Integrity of Materials and Structures, RWTH Aachen University, Intzestr. 1, D-52072 Aachen, Germany; Division of Gastroenterology, Hepatology and Endoscopy, Brigham and Women’s Hospital, Harvard Medical School, Boston, MA 02115, USA; The Department of Integrity of Materials and Structures, RWTH Aachen University, Intzestr. 1, D-52072 Aachen, Germany; David H. Koch Institute for Integrative Cancer Research, Massachusetts Institute of Technology, Cambridge, MA 02139, USA; Division of Gastroenterology, Hepatology and Endoscopy, Brigham and Women’s Hospital, Harvard Medical School, Boston, MA 02115, USA; Department of Mechanical Engineering, Massachusetts Institute of Technology, Cambridge, MA 02139, USA; David H. Koch Institute for Integrative Cancer Research, Massachusetts Institute of Technology, Cambridge, MA 02139, USA; Division of Gastroenterology, Hepatology and Endoscopy, Brigham and Women’s Hospital, Harvard Medical School, Boston, MA 02115, USA; Department of Mechanical Engineering, Massachusetts Institute of Technology, Cambridge, MA 02139, USA; David H. Koch Institute for Integrative Cancer Research, Massachusetts Institute of Technology, Cambridge, MA 02139, USA; Division of Gastroenterology, Hepatology and Endoscopy, Brigham and Women’s Hospital, Harvard Medical School, Boston, MA 02115, USA; Department of Mechanical Engineering, Massachusetts Institute of Technology, Cambridge, MA 02139, USA; Division of Comparative Medicine, Massachusetts Institute of Technology, Cambridge, MA 02139, USA; David H. Koch Institute for Integrative Cancer Research, Massachusetts Institute of Technology, Cambridge, MA 02139, USA; Department of Emergency Medicine, Brigham and Women’s Hospital, Harvard Medical School, Boston, MA 02115, USA; The Fenway Institute, Boston, MA 02115, USA; David H. Koch Institute for Integrative Cancer Research, Massachusetts Institute of Technology, Cambridge, MA 02139, USA; Division of Gastroenterology, Hepatology and Endoscopy, Brigham and Women’s Hospital, Harvard Medical School, Boston, MA 02115, USA; Department of Mechanical Engineering, Massachusetts Institute of Technology, Cambridge, MA 02139, USA

**Keywords:** biomonitoring, Kirigami, laser-induced graphene, strain sensing, wearable bioelectronics

## Abstract

Wearable sensors are transforming our capacity to monitor a broad range of activities for recreation and health purposes. Developing low-cost personalized sensors on a range of materials could enable broad applicability irrespective of the material substrate. Here, the methods of fabrication, characterization, and application of kirigami graphene strain sensors are described. The dynamic range of these sensors is characterized, showing that the kirigami structure enhances application-specific device performance, demonstrating that this strategy is applicable to a range of materials. We apply this strategy to develop personalized sensors for a variety of measurement frequencies and biological phenomena including evaluation of abdominal distention and respiration in a pig model, as well as human heart rate measurement, limb actuation, and hand gesture interpretation in human volunteers. Through these experiments, we show that this low-cost strategy for customized graphene sensors can be broadly applied across a range of consumer and health applications.

## 1. Introduction

Materials and concepts for flexible/wearable electronics have been at the forefront of medical device development in the past decade.^[1,2]^ These systems play a critical role in collecting biological and physiological data ranging from heart rate^[3]^ and respiration rate,^[4]^ to chemical analytes from perspiration.^[5]^ Increasing interest in precision medicine motivates exploration of sensors and devices which can be customized to the particular needs of the patient or disease state.^[6,7]^ Rapid prototyping methods such as 3D printing have contributed significantly to healthcare by allowing the fabrication of personalized dosage forms,^[8]^ medical devices,^[9]^ and models^[10]^ to individually accommodate a patient’s physiology. However, within the context of wearable sensors for healthcare, rapid prototyping has remained limited as most devices require numerous steps for device fabrication,^[1]^ which increase costs and decrease adoption in the clinical setting.

One low-cost fabrication method that has received increasing interest is the process of laser-induced or engraved graphene (LIG), under which the surface of a planer carbon source is converted via a photothermal process, to porous conductive graphene.^[11,12]^ Requiring only the use of a laser and a thermally stable carbon source, this process has been used for the basis of a wide range of flexible sensor concepts including mechanical sensors for sensing, electrothermal actuators, and electrochemical sensors for sensing analytes in sweat^[13,14]^ and gastrointestinal fluids.^[15]^ Within the context of strain sensing, a variety of graphene-based sensors have been developed primarily relying on the piezoresistive properties of the graphene, where a target deformation can be measured via a change in conductivity.^[16,17]^ Piezoresistive graphene strain sensors fabricated from LIG processes typically have 2D planar structures given the requirements of uniform planar source materials,^[18,19]^ which limit their capability to fit surface curvatures and accommodate more complex mechanical loading modes. In particular, strain sensing has immediate applications in healthcare as a tool to diagnose specific muscle group dyssynchrony from illness and assess progress in rehabilitation protocols over time.^[20]^

Kirigami patterns are a demonstrated strategy for adapting 2D structures to 3D configurations through the introduction of repeating cut patterns, which enhance material flexibility and allow accommodation of the material to non-Gaussian curvatures.^[21–23]^ Several design motifs have incorporated kirigami into the device substrate to enhance device flexibility or deformability, thereby improving their real-world application in measuring movement in several dimensions.^[24–26]^ Additionally, kirigami has been incorporated in systems including artificial muscle^[27]^ and pressure sensors,^[3,28]^ showing the versatility of this device fabrication strategy. However, existing embodiments of kirigami sensors are usually application specific, requiring adjustment of kirigami structures which inhibit rapid prototyping. Moreover, they usually require multistep fabrication processes,^[3,29]^ for the deposition of conductive traces, LIG conversion, and transfer or patterning on top of a kirigami substrate, which significantly increase fabrication, design iteration time, and cost.

**Figure 1.**
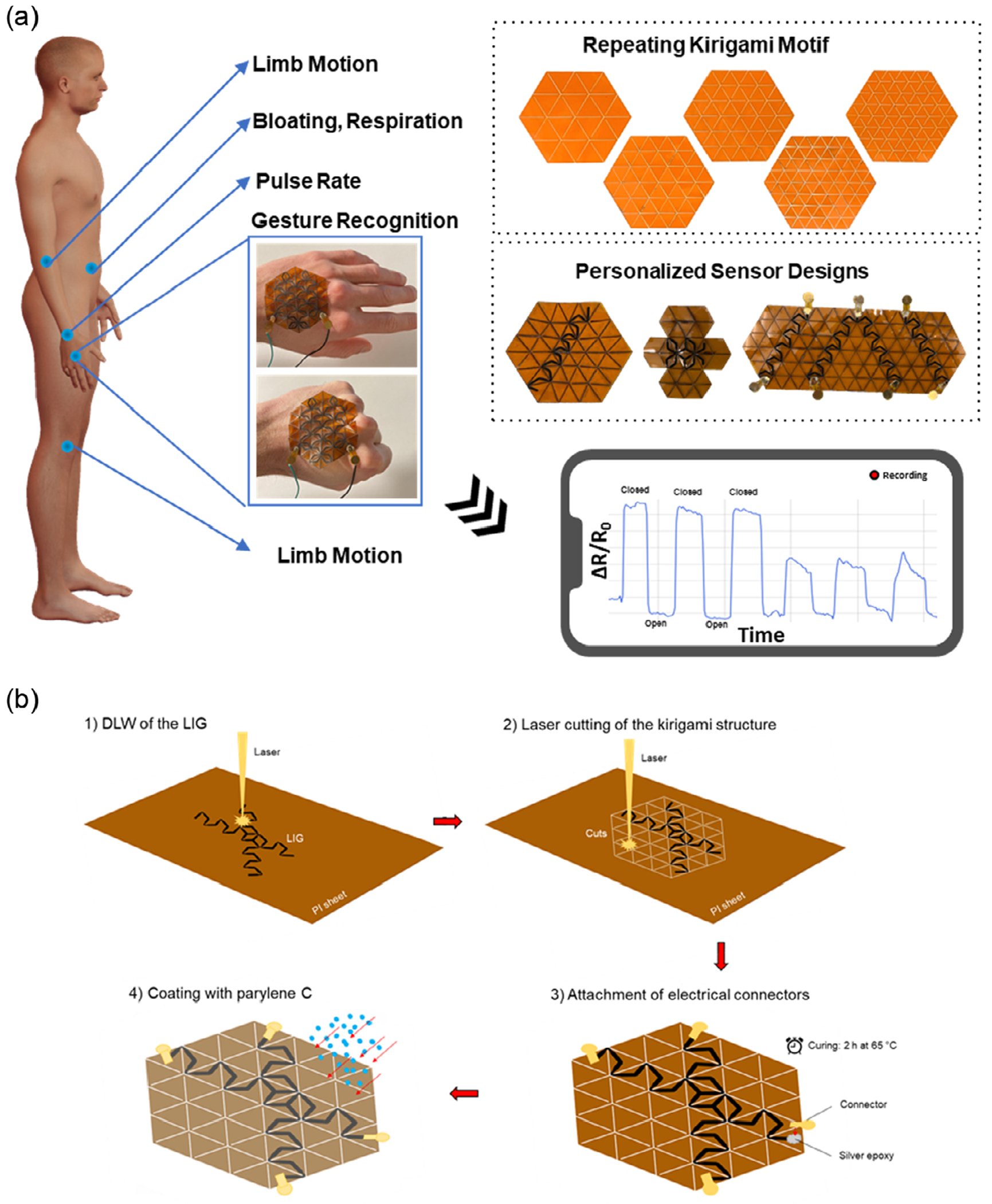
Device schematic and fabrication process. a) Illustration of use cases for strain sensors on the human body and exemplary strain resistance measurements when attached to the hand back. b) Schematic of the fabrication process of the stretchable strain sensor in four steps. 1) Direct laser writing of the laser-induced graphite on the polyimide sheet, 2) laser cutting of the kirigami structure, 3) attachment of the electrical connectors using silver epoxy, 4) coating of the sensor with a ≈2 μm parylene-C layer, if needed for surface passivation.

Here we demonstrate a low-cost and large-area fabrication of kirigami-supported LIG strain sensors with modular triangular unit cell design which can be used for a variety of human motion detection applications (Figure 1a, S1, Supporting Information). The sensors contain a sheet that is cut to a kirigami pattern, and selectively converted to piezoresistive LIG, both in a single-fabrication step using a lab-scale CO_2_ laser cutter. The key advantage of this fabrication process is its simplicity wherein the fabrication of the kirigami structure and LIG is conducted on the substrate with a single tool without need for transfer of LIG or addition of an elastomeric layer as demonstrated in prior work in the field.^[30–32]^ This reduces costs and facilitates fabrication in a clinical, point-of-care setting, allowing the rapid personalization of the sensor to the needs of a patient.

Moreover, this strategy can be widely applicable to a range of length scales and geometries by extension of the unit cells, and materials that can exhibit LIG conversion, including aromatic polymers and paper precursors.^[11,12,17,19]^ We use finite element analysis (FEA) modeling and experiments together to demonstrate that rational design of graphene traces with respect to kirigami cuts can preferentially sensitize devices to mechanical loading modes, allowing differentiation of a variety of human motions. Moreover, the kirigami vertices can be shifted as needed to better accommodate the target surface structure or application. Using these insights, we design a variety of single- and multichannel sensor structures and use them to detect motions including gastric distension and respiration rate in a large animal model as well as limb actuation, heart rate, and hand gestures recognition in wearable applications.

## 2. Results and Discussion

### 2.1. Device Fabrication Process and Design

To realize our device concept, we selected a kirigami pattern with a cut motif that creates triangular unit cells, previously reported to be able to approximate a number of surfaces.^[23]^ FEA simulations indicate that this design exhibits capability for lower maximum von Mises stress (σv, 66.05 vs. 1569 MPa) when undergoing expansion in comparison to alternating cut patterns used for sensors in literature^[29,33,34]^ (Figure S2a, Supporting Information), due to capability of in-plane rotation of the polygonal units.^[23,35]^ Additionally, in contrast to the hierarchical square cut patterns, the triangular unit cell pattern exhibits less peak stress during isotropic loading (Figure S2b, Supporting Information). Moreover, the triangular unit cells can provide sufficient area for routing of LIG traces and tracks.

**Figure 2.**
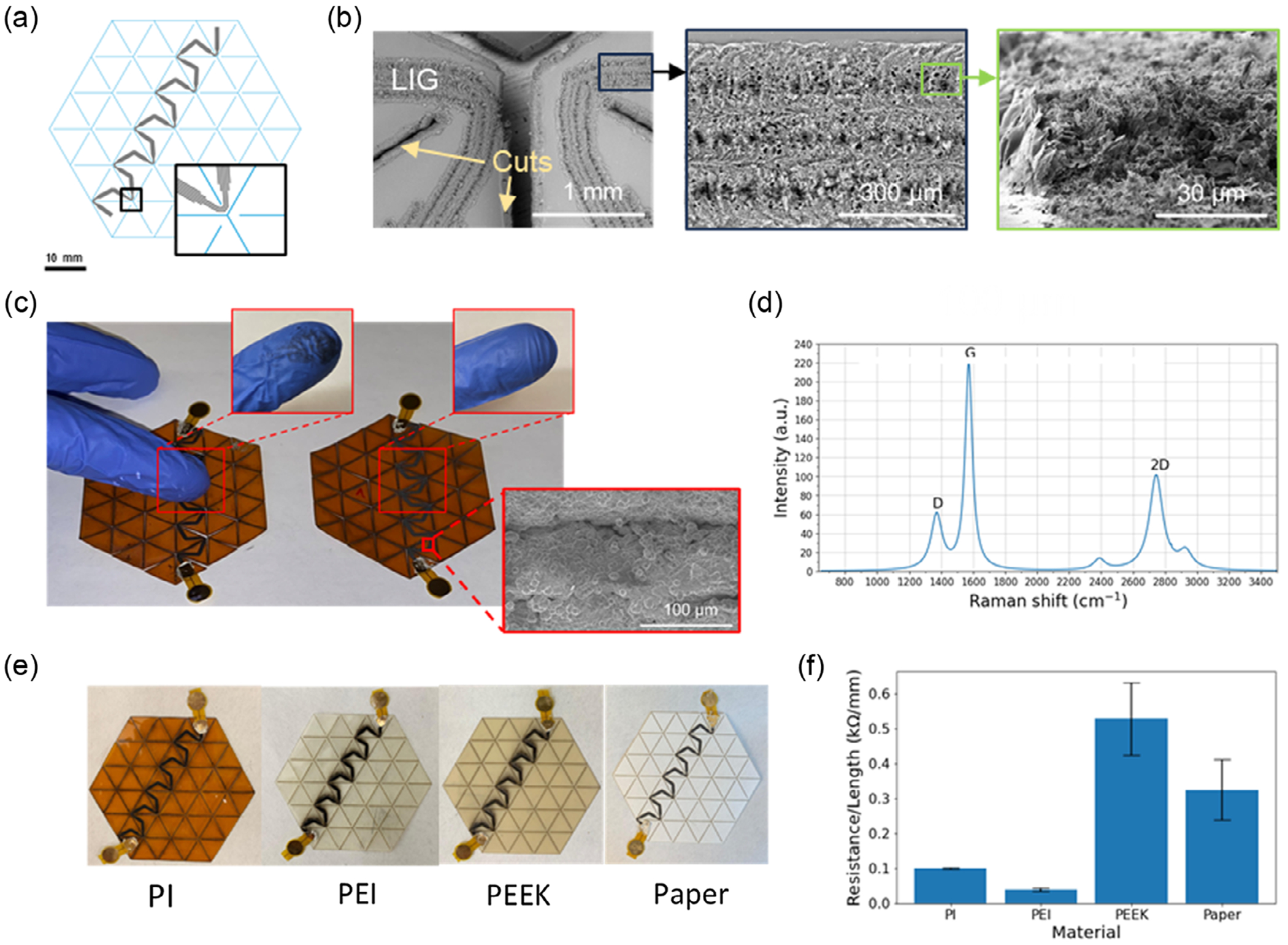
Device and materials characterization. a) Design of kirigami pattern with 54 units and the laser-induced graphene trace. Blue lines—designated cut vectors, black lines—designated LIG vectors. Inset: close up of graphene trace design near a kirigami vertex. b) SEM images of the LIG trace consisting of 3–7 offset lines (three on the hinges and seven across the wider triangle surface). The first two SEM images were taken from a top view, and the third SEM image was taken in a side view of ≈30°. c) Abrasion test of the LIG trace with uncoated (left) and coated (right) devices showing the protection of the porous graphite through the parylene-C coating. SEM image of the coated trace. d) Raman spectrum of the LIG trace shows three prominent peaks: D peak at ≈1370 cm^−1^, G peak at ≈1570 cm^−1^, and 2D peak at ≈2740 cm^−1^. e) Comparison of the device fabrication process on polyimide (PI), polyetherimide (PEI), polyetheretherketone (PEEK), and filter paper and the f) respective electrical resistances/L of designed trace for each test material (*N* = 4 for each).

The standard fabrication process for kirigami laser-induced graphene (KLIG) sensors was first demonstrated on a 75-μm polyimide film (Kapton, Dupont) using a 54 triangle design, in a hexagonal shape with a 30 mm-edge length. Traces of LIG are patterned, followed by laser cutting (Figure 1b, S1, Supporting Information). Adjustments between defocus, power, and speed were used to switch between cutting and LIG induction (Experimental Section). Initial experiments showed that rastering a defined area yielded distinct graphene domains while vector lines were able to form continuous connected LIG (Figure S3, Supporting Information). Consequently, the graphene traces were designed as a set of 3–7 parallel lines with a pitch of 0.15mm (Figure 2a), depending on the required thickness for sensing and routing. In routing areas, trace thickness was increased to decrease resistance/contribution to the piezoresistive effect, while in sensing areas (the vertices), trace thickness was decreased to enhance contribution to the overall resistance. For motion sensing measurements, overall trace resistance was designed to be ≈5 kΩ to be within the dynamic range of the measurement electronics (Experimental Section). Scanning electron microscopy confirms the formation of porous structures in the exposed areas (Figure 2b) and Raman spectroscopy confirms the formation of disordered graphene (Figure 2d) as seen from the presence of the D and 2D peaks, similar to literature reports.^[12]^ Following cutting, electrical connections are attached via silver epoxy at desired points of the LIG traces (Figure 1b). Bonding can be done to either a gold pad on a flexible substrate (Figure 2c) or a standard flat flexible cable connector (Figure S4, Supporting Information), shown to demonstrate easy interfacing to commercial or custom-designed measurement electronics. For applications where mechanical motion may cause surface abrasion of the sensor, the surface can be protected by coating with a 2 μm layer of parylene-C (Figure 2c, S5, Supporting Information), which provides the LIG protection detachment or transfer due to contact with other surfaces. Imaging indicates that the parylene-C layer provides a conformal, continuous polymer layer (Figure S5, Supporting Information) over the porous graphene which may be the cause of the additional mechanical resilience. Moreover, this process is adaptable to other materials that have sufficient aromatic content to generate LIG including polyetherimide (PEI), polyether ether ketone (PEEK), and filter paper (Figure 2e), where repeatable conductivities/lines (Figure 2f, *N* = 4 for each sample) were obtained for each material-specific optimized laser condition. These materials were selected to demonstrate that the KLIG process could potentially be applicable in a wide range of contexts: PEI is commonly used as a fused filament fabrication 3D printer resin, PEEK is used as a high-mechanical-strength thermoplastic, and filter paper can be readily used in systems that require biodegradability. Further experiments focused on the use of the polyimide backing material as it is widely used in biomedical devices, relatively low cost, and is known to have excellent long-term biocompatibility.

The key advantage of this fabrication process is that it only requires the use of a CO_2_ laser cutter and an optional step involving parylene-C coating. Additionally, using this process, a variety of geometries can be fabricated via changing the number of unit cells and/or moving the vertex points of the cuts, where the limitation is only due to the required hinge width and yield stress of the material at the target desired deformation. This facile fabrication allows for rapid prototyping to generate personalized sensors for a specific use-cases, which can vary depending on the particular surface geometries of the particular application.

### 2.2. Mechanical Testing of KLIG Sensors

**Figure 3.**
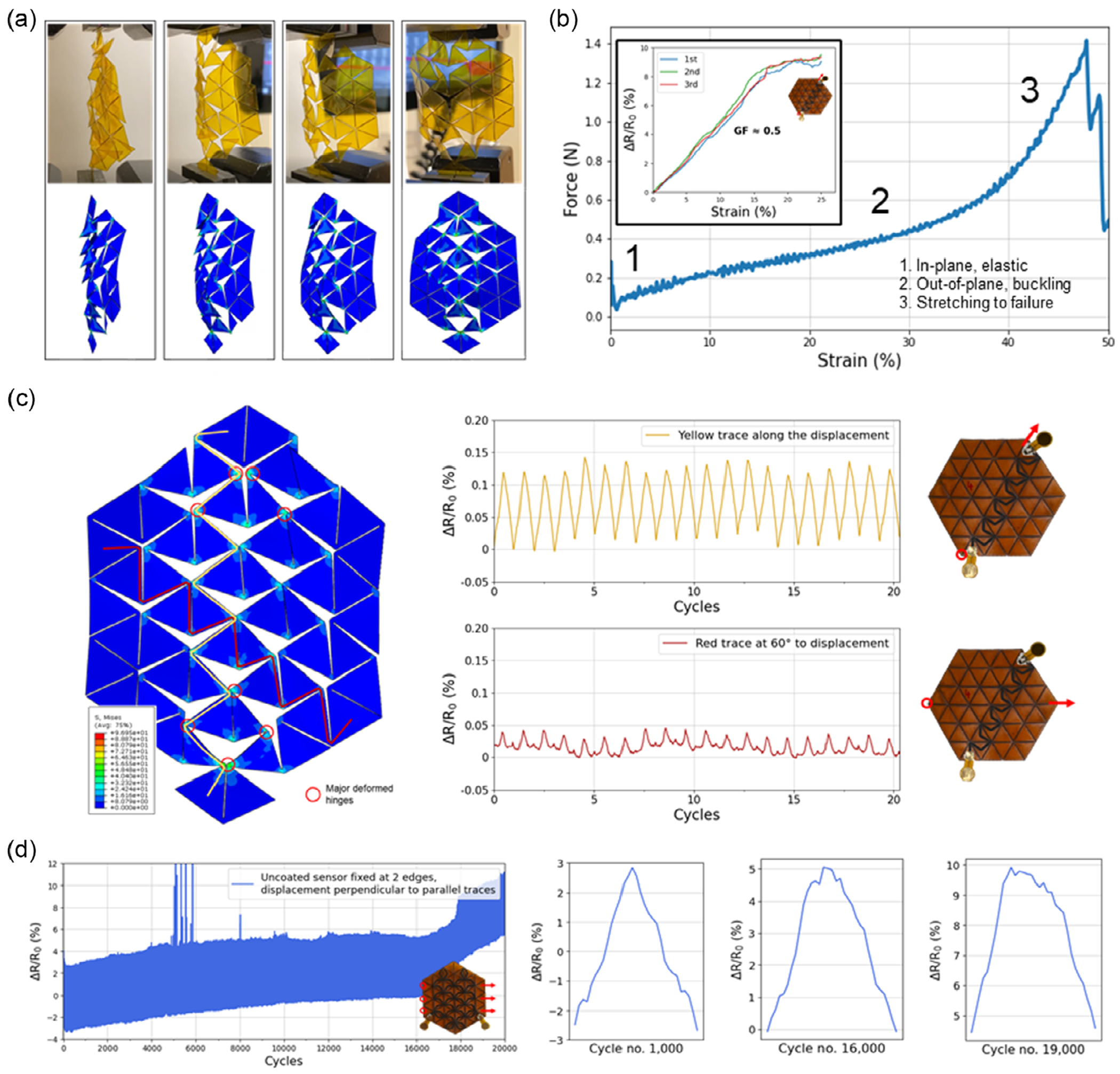
Mechanical loading and strain-sensing capability of the kirigami sensor prototype. a) Comparison of the predicted deformations of the sensor (25 μm and 54 units) by the FEA simulations with the real deformations from the mechanical testing (displacement of 7.5 mm). b) Tensile test of the kirigami pattern showing stages of elastic, buckling, and plastic response. Inset: Gauge factor measurement of graphene trace under a parallel tensile load. c) Cyclic loading and strain resistance measurement of the device (75 μm and 54 units) and 20 out of 500 cycles with 1) the trace along the displacement and 2) the trace at 60° to the displacement. FEA simulation indicates higher deformation of the trace along the displacement, also resulting in a higher resistance change and gauge factor. d) Cyclic loading and strain resistance measurement of the device (75 μm, 54 units, uncoated with trace spanning the face of the pattern) for 20 000 cycles with fixed edges and a displacement of 7.5mm perpendicular to the parallel traces and representative cycles at the beginning (cycle no. 1000), before the steep resistance increase (cycle no. 16000), and at the end (cycle no. 19000).

Following validation of the fabrication process workflow, we explored the fundamental mechanical properties and sensing capability of the KLIG sensor. The deformation and buckling of the kirigami pattern agrees well with simulations (Figure 3a), where stress during loading is focused on the vertex hinge points. When a tensile load is applied to the two corners of the design, it first undergoes elastic, in-plane stretching, followed by out-of-plane buckling, and then ultimately, stretching and failure at the hinge points at around a strain of ≈45% (Figure 3b). Fabrication of a graphene trace along the design followed by mechanical testing confirms the effectiveness as a piezoresistive sensor with a gauge factor of ≈0.5 up to up ≈20% in during lateral stretching applied from the corner of the structure (Figure 3b, inset). Sensor response time was instantaneous within the resolution of the measurement electronics (1 khz). Given simulated stress at vertex points, designs were then explored where LIG traces were placed either along or at a 60° bias to the major deformed vertices of the kirigami structure (Figure 3c) under cyclic loading. Measurement of resistance change shows an approximately fourfold increase in relative resistance change for the design which maximized LIG traces along the kirigami vertices with peak strains (≈10 peak strain vertices) versus an orthogonal trace (≈3 peak strain vertices). Additionally, cyclic loading of another KLIG sensor shows stable performance for up to 16 000 cycles, indicating the robustness of the sensors (Figure 3d). Testing of parylene-C coated sensors shows enhanced stability of the KLIG sensor performance to past 20 000 cycles (Figure S5, Supporting Information), at the expense of a reduction in the sensor gain, which could be valuable for applications that require a larger numbers of cycles.

The aforementioned mechanical testing shows a generalized strategy for placement of LIG traces along the kirigami structure to selectively target or suppress measurement of a loading mode, allowing for application specific design adaptations. As the simulations confirm that the stress is focused on the kirigami vertexes and majority of the LIG trace does not deform during stretching, portions of the traces on the triangular faces primarily are used for routing and can be widened in certain designs to decrease overall resistance and improve sensor gauge factor depending on sensing requirements. Moreover, this asymmetric stress focusing enables rational design of LIG trace location placement to target or exclude certain loading modes depending on sensing requirements, via passing through or avoiding vertices where stress is localized.

### 2.3. KLIG Sensors for Varied Use Cases

To demonstrate the generalizability of our fabrication strategy to prototype strain sensors, we developed KLIG sensors to measure a variety of motions for medically relevant applications and tested these sensors either on animal model or human volunteers as appropriate. We selected several different use cases that would exhibit signals of different time scales, and mechanical deformations, and adjusted the sensor design as needed to match to specific measurement via modification of LIG traces, kirigami vertices, and fixation to the substrate. Sensor fixation was achieved through use of adhesives, tapes, or mechanical pressure depending on the application as detailed later, to ensure effective mechanical coupling between the area of interest and the KLIG sensor.

**Figure 4.**
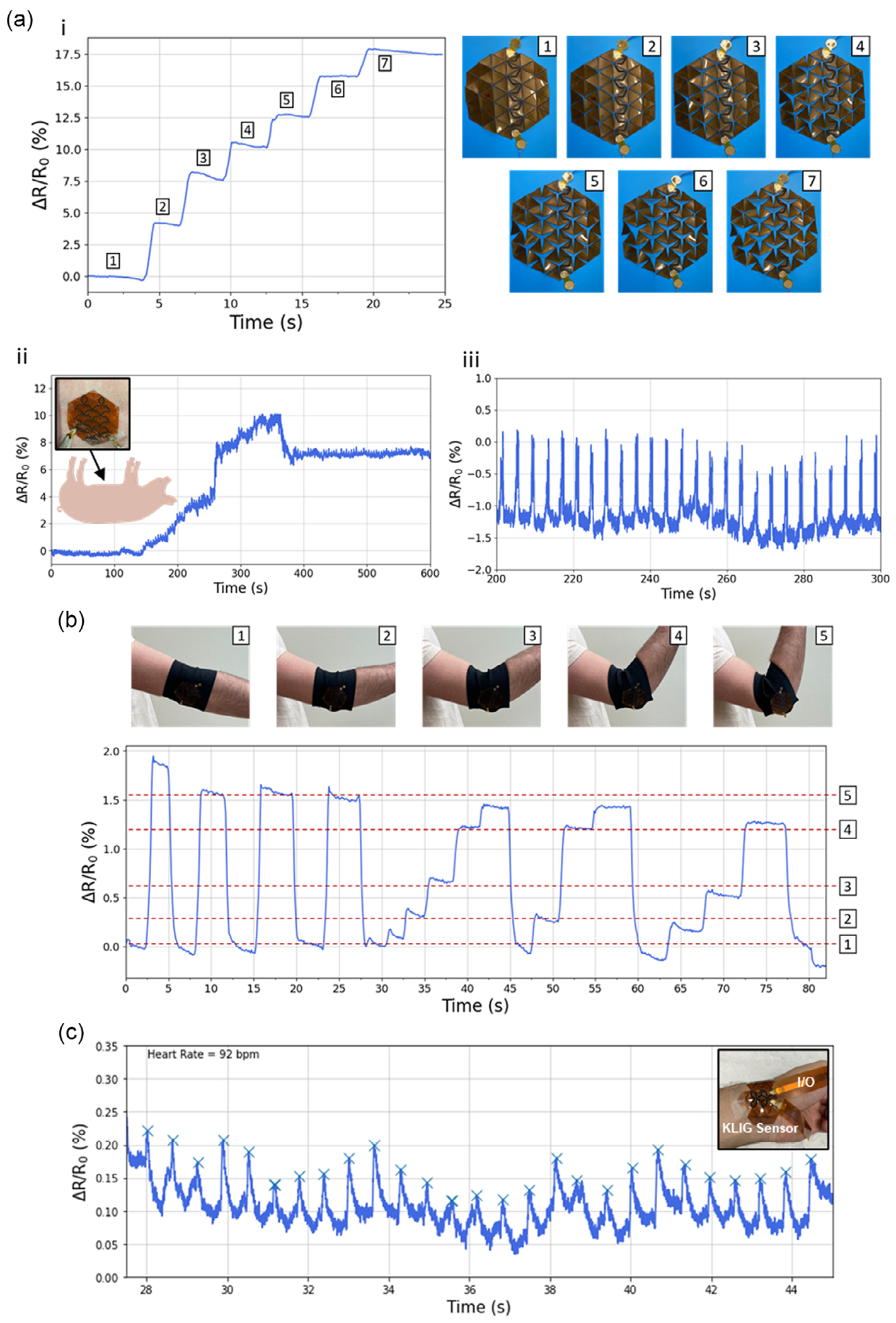
Applications of the stretchable strain sensor. a) Bloating and respiration detection with the device. i) Device fixed to balloon and indicating resistance increase with each expansion of the balloon. ii) Simulation of gastric bloating. Sensor attached to porcine abdomen and indicating resistance increase with simulation of gastric bloating via endoscopic insufflation. iii) Respiration monitoring showing increasing resistance with each breath from the KLIG sensor attached on the porcine abdomen. b) Human motion monitoring. Real-time motion monitoring for different bending levels of the elbow. Sensor showing stepwise resistance increase with every degree of flexion (from ≈1—straight to 5°–90°). c) Heart rate measurement using KLIG sensor, with peak tracking indicating 92 bpm in agreement with commercial fingertip pulse monitor. Inset: Image of KLIG sensor on wrist for pulse measurement, elastic sleeve not shown for photo clarity.

We started by exploring the simplest loading mode that is an isotropic expansion, where the KLIG sensor was bonded to a rubber balloon (Figure S6a, Supporting Information) by application of an adhesive to the triangular faces of the kirigami pattern. Either a single LIG trace across the center of the kirigami pattern (Figure 4ai) or spanning the whole surface area of the kirigami pattern (Figure S6b, Supporting Information) can be placed to detect varied states of inflation corresponding to increasing resistance during differing degrees of expansion. The similarity in performance between these two trace designs confirms that isotropic expansion equally stresses all vertices in the kirigami pattern. This type of deformation can be used to monitor volume expansion in the form of abdominal distension, gastric volume, bladder distension, or other similar medical conditions. We model this by attaching the kirigami sensor to an abdominal binder on the external abdomen of an anesthetized Yorkshire swine (Figure S7, Supporting Information). Insufflation of the stomach during endoscopy was used to simulate gastric bloating or distension,^[36]^ and a corresponding change in the measured resistivity was measured (Figure 4aii). When insufflation was halted, there was a slight decrease in the resistivity which we attribute to viscoelastic tissue relaxation followed by a stable resistance measurement of the stomach in the distended state. Moreover, during these experiments, we observed that if the kirigami sensor was attached tightly to the abdominal surface, periodic peaks ≈15.6 min^−1^ could be observed. This frequency correlates with the 16 breaths min^−1^ measured on the vital signs monitor, indicating that the KLIG sensor can detect abdominal deflections due to animal respiration (Figure 4aiii).

As an example of motion detection, we continued by designing an KLIG sensor to measure limb actuation at the elbow joint in a study volunteer. The kirigami vertices were adapted via shifting of the pattern vertices to fit the curvature of the subject’s elbow and then bonded in the corners to an elastic wearable neoprene sleeve (Figure S8c, Supporting Information). The KLIG sensor is shown to be sensitive to a variety of degrees of limb actuation (Figure 4b). When the elbow was bent to a new position, we see an increase in resistance followed by a small decrease in resistance, which we attribute to relaxation of the neoprene sleeve in the new configuration.

To further demonstrate the adaptability of the KLIG sensor, we designed a sensor to measure the heart rate of a healthy human volunteer. A modified KLIG sensor (Figure S9, Supporting Information) was attached to the wrist of a subject using a medical tape and an elastic wrist wrap. The resistance of the LIG trace was measured over ≈30 s (Figure 4c). Filtering with a low-pass filter using a third gradient sum of absolute differences approach^[37]^ reveals spikes with a frequency of 86 min^−1^ which was consistent with heart rate extrapolated from the oxygen saturation waveform obtained by a pulse oximeter (Santamedical, CA, USA). The capability of the KLIG sensor to measure pulse indicates that the kirigami structure is compatible with measurement of higher-frequency, low-amplitude events.

**Figure 5.**
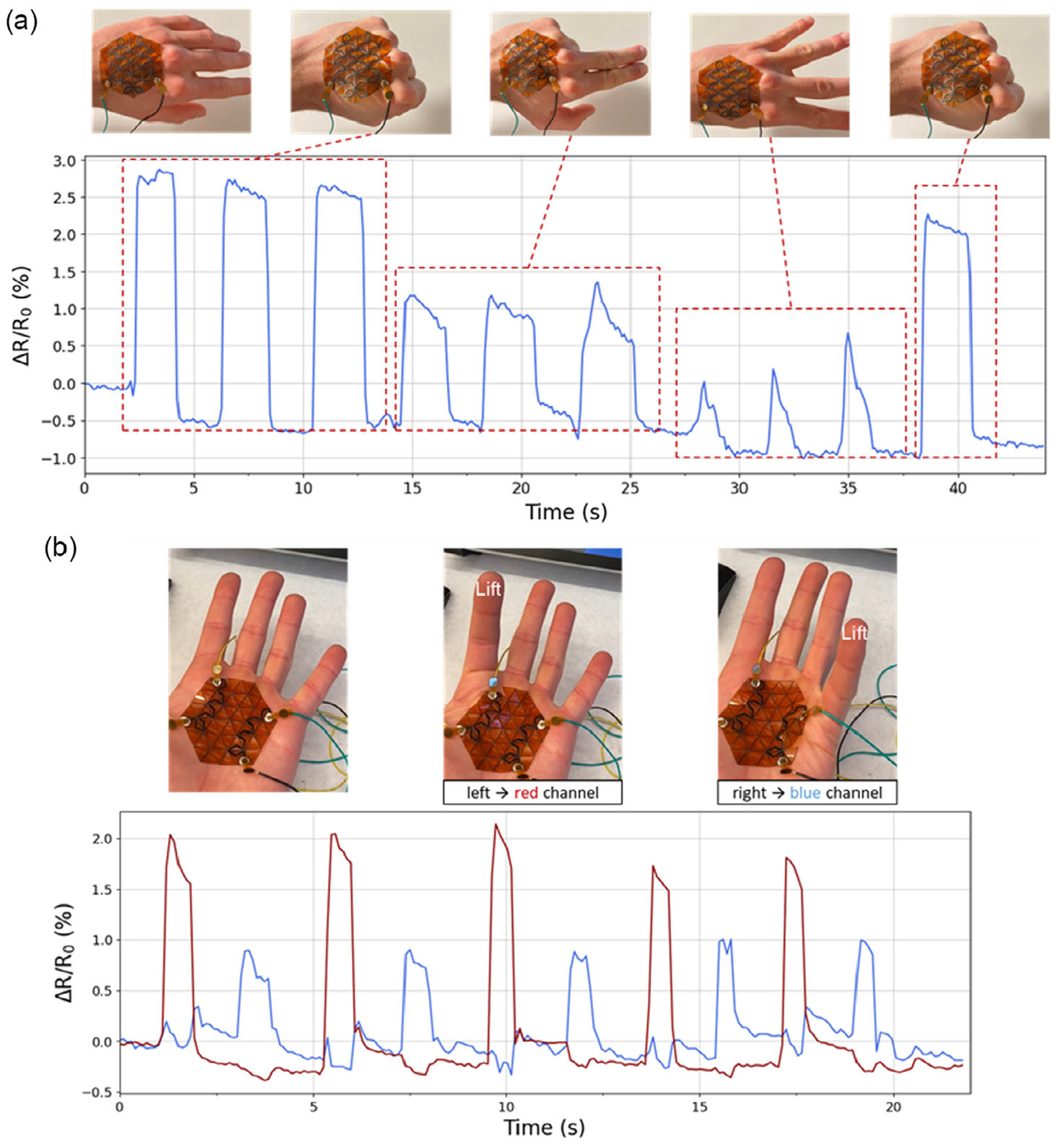
Hand gesture recognition using KLIG sensor. a) Real-time motion monitoring of different hand gestures using a KLIG sensor. Device was able to monitor different hand and finger movements. Baseline drift between gestures is due to variations in the neutral hand position (relaxed open palm) of the subject during performance of the gesture pattern. b) Real-time motion monitoring of different finger movements with a two-channel sensor consisting of two parallel traces monitoring index and small finger movements.

Additionally, we demonstrated that the KLIG sensor can obtain useful information under conditions where the loading mode cannot be fully decoupled. Gesture detection is an area of growing interest^[38]^ for human–machine interactions. However, hand gestures are typically compound motions that recruit a range of muscle groups and can generate a loading mode that cannot be fully isolated in a single trace. To address this, we instead designed a single-LIG trace that spans the surface area of the dorsum aspect of the hand. Following fixation to the hand using medical tape, we measured the resistance during a range of different performed gestures. Gestures such as the open palm, fist, two fingers or four fingers can still be differentiated from the waveform shapes of the measured resistance (Figure 5a). We attributed this to a range of muscular actuations required to achieve each gesture, which varies the order and number of vertices deformed, which generates these relative resistance waveforms. Further work into this target application could also focus on generating a personalized dataset for each subject under various gesture conditions to classify and differentiate the measured signals.

Finally, as a proof of concept for multiplexed measurements using KLIG sensors, two parallel traces on the edge of the kirigami structure were then designed for detection of digital actuation of the index and pinky fingers of a subject. The KLIG sensor was attached to the palm of the subject with medical tape at the six corners of the hexagonal pattern, and then resistance in both channels was measured during lifting of either the index or pinky finger (Figure 5b). The left channel was responsive only to the index finger actuation, while the right channel was responsive only to the pinky finger actuation. These independent measurements show the capability of the rational design of the kirigami structures which both span a large area but also are able to isolate target loading modes for sensing that primarily deform a subset of the vertices.

## 3. Summary and Conclusion

We demonstrated the generalized strategy for rapid prototyping of personalized strain sensors using a kirigami-inspired design coupled with LIG. The design is modular, scalable, and adaptable to a variety of uses via selective placement of kirigami cuts and sensing LIG. The fabrication process can be accomplished easily with a standard CO_2_ laser cutter and can be conducted on a range of different materials, so long as they have sufficient aromatic content to generate graphene and can be cut using the laser cutter. The sensor is stable for up to 16 000 cycles and can be further extended by parylene-C coating to 20 000+ cycles in applications that require long-term performance. Moreover, the designs can be easily scaled and adapted to a wide range of applications to specifically detect a target loading mode which could be related to motor actions or abdominal distension. The simplicity of the fabrication enables the use of this method to rapidly produce low-cost, easily replaceable, personalized strain sensors fit to the specific needs of a patient.

In the future, this process could be applied to manufacture sensors for a variety of clinical applications where conformation to an individual patient’s anatomy is critical, such as monitoring limb and joint actuation during physical therapy, wearable gesture interfaces for human computer interfaces, and monitoring changes in abdominal distension in individuals undergoing weight loss treatment. This work could be further expanded in the future using computer vision mapping of patient morphology and machine learning/computer automated design to facilitate and rapidly iterate on sensor and kirigami design, to create a large library of easily accessible sensor morphologies. Finally, our method could also be expanded past materials that can be directly converted to LIG, via coating of substrates with a precursor suitable for LIG conversion while laser cutting is used to generate the kirigami pattern. In summary, our KLIG process can generate wearable personalized sensors which we envision will play an important role in realizing personalized healthcare, ultimately toward the improvement of patient diagnosis and monitoring.

## 4. Experimental Section

### Device Fabrication:

The sensor prototypes were fabricated using a CO_2_ laser cutter in two steps. First, the porous graphene was generated by direct laser writing of the base material (Kapton, 75 μm thickness). Second, the laser was used to cut the kirigami pattern into the substrate. A CO_2_ laser cutter system (VLS3.60DT, Universal Laser Systems) with a 50W laser source and a wavelength of 10.6 μm was used to perform the direct laser writing process and cutting of the material.

Besides the laser power, the scan rate of the laser beam (maximum speed: 400mm s^−1^), the pulse rate (maximum: 1000 PPI, pulses per inch), and the focus of the laser beam by varying the height in the z-direction can be adjusted. The input files for the laser cutting machine (e.g., DXF and DWG files) were generated with the computer-aided design (CAD) software SolidWorks (Dassault Systems) or Adobe Illustrator (Version 25.3.1 (64-bit), Adobe). Parameters used for each material and process are detailed in Table 1; PI, PEI, and PEEK were purchased from McMaster-Carr, and Whatman filter paper was purchased from Sigma-Aldrich.

Silver conductive epoxy adhesive (Type 8330S, MG Chemicals) was applied between the trace and the connector and cured afterward for 2 h at 65 °C. In the case of multiple traces that are close together, a stencil mask was produced from 125-μm thick Kapton to place the silver epoxy precisely. For devices that required passivation, a parylene coating system (PDS 2010 Labcoater, Specialty Coating Systems) was used. Electrical connections were protected with a tape, and the sensors were put into the coater and an ≈2 μm layer of parylene-C was deposited on the surface of the sensor.

### SEM Characterization:

For the scanning electron microscopy (SEM), the specimens were mounted using a double-sided carbon tape and were imaged using the high-vacuum secondary electron detector from the Hitachi FlexSEM TM-1000 II. Low-voltage imaging (1.5–2 kV) was used in substitution of a conductive coating but also provided high surface detail. The SEM images were captured using frame integration to average out surface charging artifacts. When charging artifacts became unmanageable, variable-pressure mode (50 Pa) was used with the backscatter electron detector at 7 kV.

### Raman Characterization:

Raman microscope (XploRA, HORIBA France SAS) with a 405-nm excitation laser was used. Areas containing LIG were located with the attached optical microscope and the LIG was then scanned for 10 s and the integrated software was used to fit the peaks of the Raman spectrum.

### Electrical and Mechanical Characterization:

Kirigami structures were mounted on a universal testing machine (Model 5943, 1 kN capacity, Instron), which was equipped with a 1 kN load cell (2580-1KN, accuracy: 0.005 N). Both tensile and compression tests until 7.5 and 10 mm displacement, respectively, each with fixed corners and edges, were conducted. Moreover, a flexural loading mode was tested with a 3D-printed bracket and stamp to penetrate the structure. Displacement rates from 10 to 20 mm min^−1^ were set.

Resistance strain measurements were carried out under the same system by connecting the sensor electrical leads to a digital multimeter (DM3058, Rigol) during stretching or compressed with the universal testing machine. The change of resistance during deformation was collected with a data logging software (UltraView, Rigol).

### Numerical Simulations:

Numerical simulations were carried out using a commercial finite-element (FE) package Abaqus 2021 (SIMULIA, Providence, RI, USA). We constructed the FE models of the different kirigami patterns and obtained the deformation behavior of the selected base pattern for modifications of the base design. The Abaqus/Explicit solver was employed for all simulations, and the Nlgeom setting was turned on by default to account for geometric nonlinearity. The parts were either designed with the Abaqus integrated CAD software or imported from SolidWorks (Dassault Systems) as an IGS file. Since plastic deformations of the kirigami structures should not occur, a linear elastic material model was used with the Young’s modulus (2.5 GPa), the Poisson’s ratio (0.34), and the density (1.42 g cm^−3^) as input material parameters. The resulting stresses were compared with the yield strength to verify that the elastic regime has not exceeded.

### Isotropic Expansion Testing:

For the measurement of isotropic extensions, the sensor was fixed to a balloon using cyanoacrylate glue and the expansion of the balloon with inflowing air was measured during balloon inflation using a bicycle pump.

### Animal Experimentation:

All animal experiments were approved by and performed in accordance with the Committee on Animal Care at MIT. Yorkshire swine were obtained from Cummings School of Veterinary Medicine at Tufts University (Grafton, MA, USA) for in vivo experiments. An animal weighing ≈85 kg was placed on a liquid diet for 24 h one day before the study. The animal fasted overnight immediately prior to the study. The animal was anesthetized using an intramuscular injection of midazolam 0.25 mg kg^−1^ and dexmedetomidine 0.03 mg kg^−1^. Following sedation, animals were placed on thermal support, and ophthalmic ointment was applied to both eyes. The animal was intubated and placed on isoflurane (2%) in oxygen while connected to a vitals signal monitor. The KLIG sensor was attached to the surface of the animal abdomen using medical tape (Fixomull) on the edges of the sensor, and then the animal abdomen was wrapped in a veterinary wrap bandage to secure the device. The sensor was connected to a multichannel digital multimeter (DAS240-BAT, BK Precision, Sefram) for data readout. Endoscopy was performed using a PENTAX EC-3870TLK (160 cm), and the stomach was insufflated with air via the endoscope to simulate abdominal distention.

### Human Motion and Pulse Detection:

The human study was approved by the MIT Committee on the Use of Humans as Experimental Subjects (COUHES, Protocol #2304000974) and performed under the auspices of MIT. Volunteers were recruited via word of mouth. Three healthy human subjects were recruited, and signed consent forms were obtained before the start of the study. A multichannel digital multimeter (DAS240-BAT, BK Precision, Sefram) was used in all studies to measure changes in sensor resistance.

For measuring the amount of stretching of the arm, the supporting material (High Elasticity Knit Elastic Band, CISONE) was first cut into the right size and then sewn together with a hand sewing machine (Handy Stitch, Royalsell). The sensor was fixed to the supporting material with double-sided adhesive tape (7139A21, McMaster-Carr). For the application of the sensor to measure motions of the hand and the fingers, the sensor was directly fixed both onto the back and the palm of the hand using medical tape (Fixomull) at the edges of the sensor. For measurement of the pulse, the sensor was fixed using medical tape and covered with an elastic wrist wrap. For all measurements, care was taken to avoid skin irritation during sensor attachment.

### Data Analysis:

Recorded data were parsed using custom code in python using pandas, numpy, matplotlib, and scipy libraries. Signal processing was conducted using the scipy signals module, and graphical representations of data were generated using matplotlib. The code that supports the findings of this study are available from the corresponding authors upon reasonable request.

## Supplementary Material

supplemental information

## Figures and Tables

**Table 1.** LIG induction parameters.

Sample^a)^	Power [%]	Speed [%]	PPI	Defocus [mm]
PI (75 μm)	1.5 (7)	3.5 (10)	750 (500)	−1.8 (0)
PEI (75 μm)	1.5 (2)	3 (6)	1000 (500)	−2.1 (−0.7)
PEEK (75 μm)	1.8 (2)	5.5 (6)	750 (500)	−1.4 (−0.7)
Filter paper	0.2 (0.8)	1.5 (3.5)	490/290 (500)	−2.1 (0)

a) Parameters in parentheses indicate laser conditions used for cutting following LIG induction. Note for the filter paper, the LIG induction step was conducted twice at succession, once at 490 PPI and followed by at 290 PPI, to avoid overheating of the paper.

## Data Availability

The data that support the findings of this study are available from the corresponding author upon reasonable request.
