# Supplementary material for: Personalized Kirigami Strain Sensors for in vivo Applications
Source: Adv Intell Syst. Author manuscript; Available in PMC 2025 Sep 27. (PMC12467391; doi:10.1002/aisy.202400886)
Supplement: supplemental information [file NIHMS2060203-supplement-supplemental_information.pdf]

WILEY-VCH

Supporting Information

Personalized kirigami strain sensors for in vivo applications

*Siheng Sean You, Max Schober, Soufyane Ben-Ayed, Sahab Babaee, Stephanie Owyang, Josh Jenkins, Peter R Chai, Giovanni Traverso**

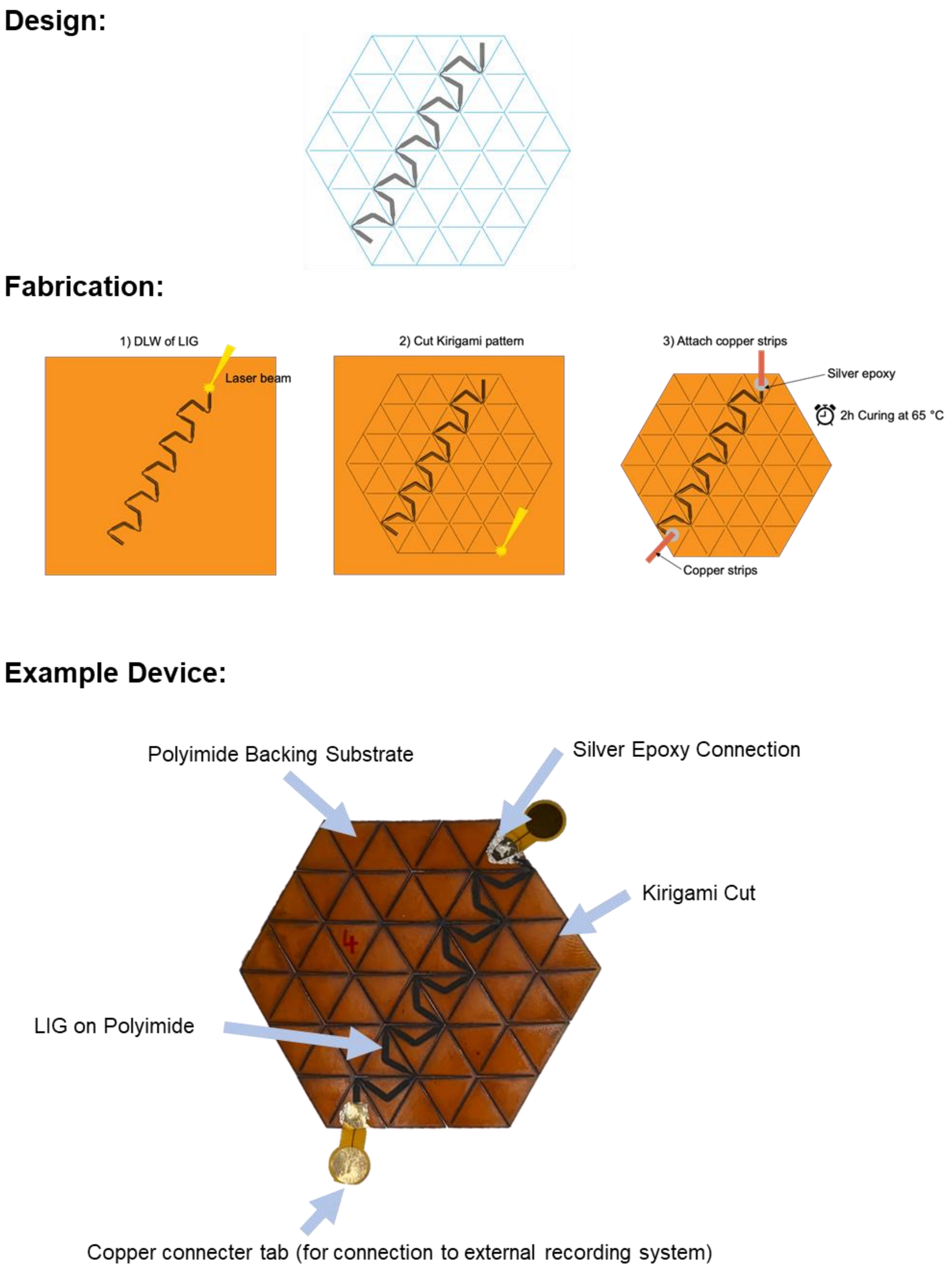

Figure S1. Design and fabrication process in a flat view, with a labeled device after fabrication.

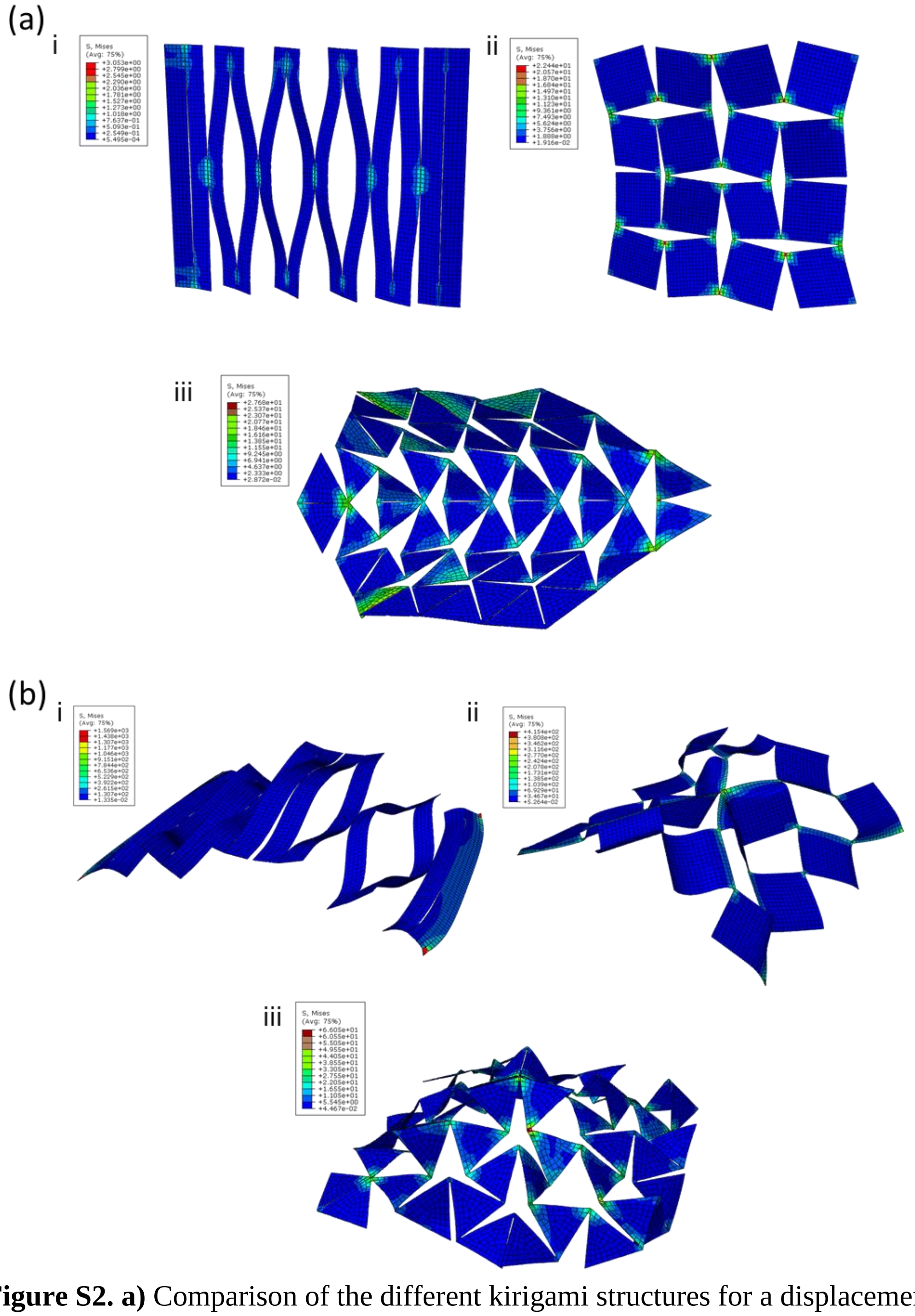

Figure S2. a) Comparison of the different kirigami structures for a displacement of 10 mm in x-direction. i) Alternating-offset-cuts structure (max. σ_v = 3.05 MPa), ii) Square fractal cuts structure (max. σ_v = 22.44 MPa), iii) Triangular units structure (max. σ_v = 27.68 MPa). **b)** Comparison of the different kirigami structures for displacements in all directions. i) Alternating-offset-cuts structure (max. σ_v = 1569 MPa), ii) Square fractal cuts structure (max. σ_v = 415.4 MPa), iii) Triangular units structure (max. σ_v = 66.05 MPa)

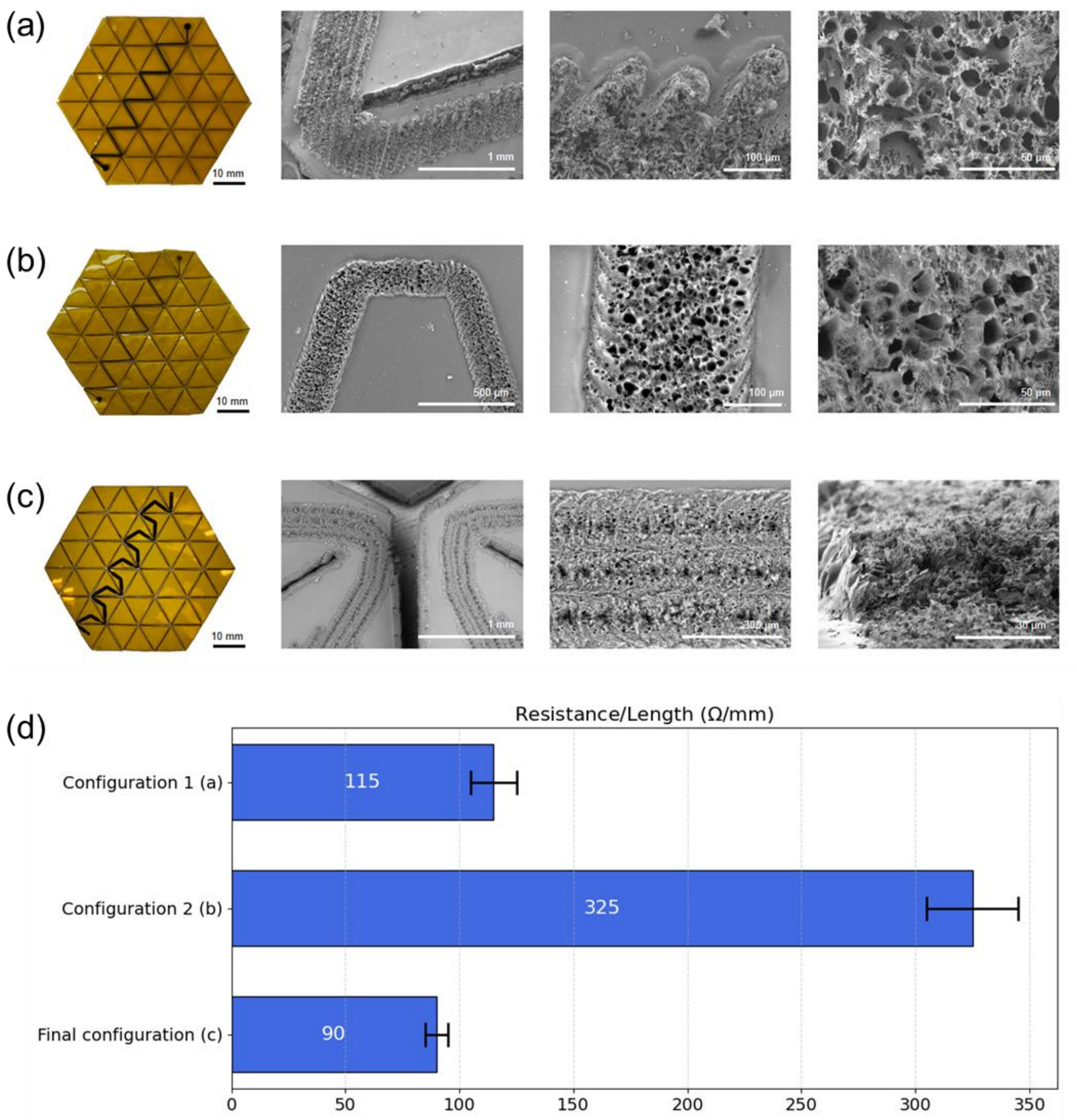

Figure S3. Photos and SEM images of the LIG traces for different laser parameter configurations and approaches. **a)** Wider trace with time-intensive raster motion setting and without defocus leading to small gaps between the LIG lines and losses in conductivity; average resistance of 115 Ω/mm (+/- 10 Ω/mm for N=3). **b)** Thinner trace with vector motion setting and defocus with increased quality and density of the LIG trace but thinness leading to conductivity losses due to a wearing of the LIG upon repeated loading; average resistance of 325 Ω/mm (+/- 20 Ω/mm for N=3). **c)** Final configuration with traces consisting of 3-7 offset lines with defocus and vector motion setting minimizing trace line resistance and time spent; average resistance of 90 Ω/mm (+/- 5 Ω/mm for N=3). **d)** Resistance / Length of each LIG trace design

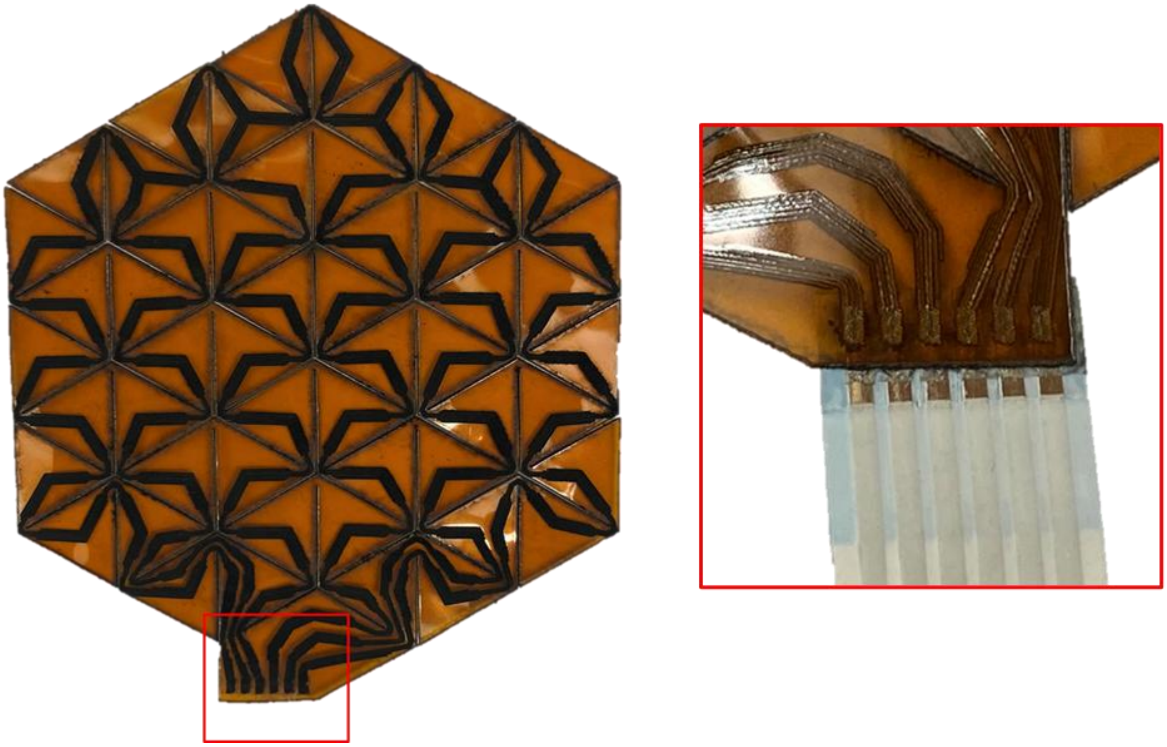

Figure S4. Bonding of KLIG to molex FFC connector using silver epoxy

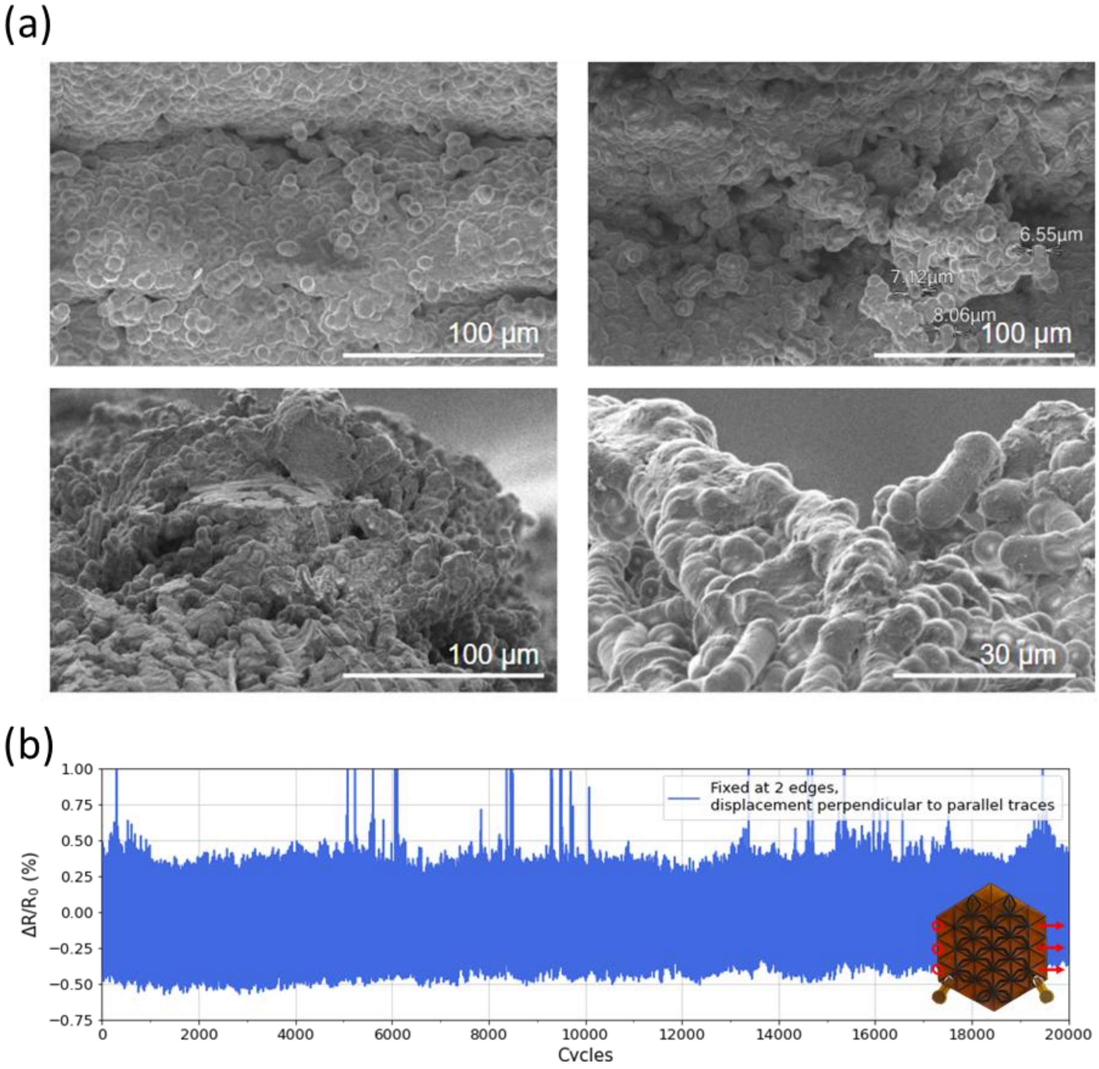

Figure S5. a) SEM images of parylene-C coated LIG traces showing a continuous polymer layer. Note the top left figure is an enlarged image of the inset from Figure 2C . **b)** Cyclic loading and strain-resistance measurement of the device (75 µm, 54 units, coated with trace spanning the face of the pattern) for 20,000 cycles with fixed edges and a displacement of 7.5 mm perpendicular to the parallel traces. The increase in cycling stability suggests that the parylene-C layer mechanically supports the graphene layer to prevent deformation/damage. We attribute the decrease in sensor gain as due to the restriction in movement of graphene domains during expansion/contraction due to the continuous parylene-C layer.

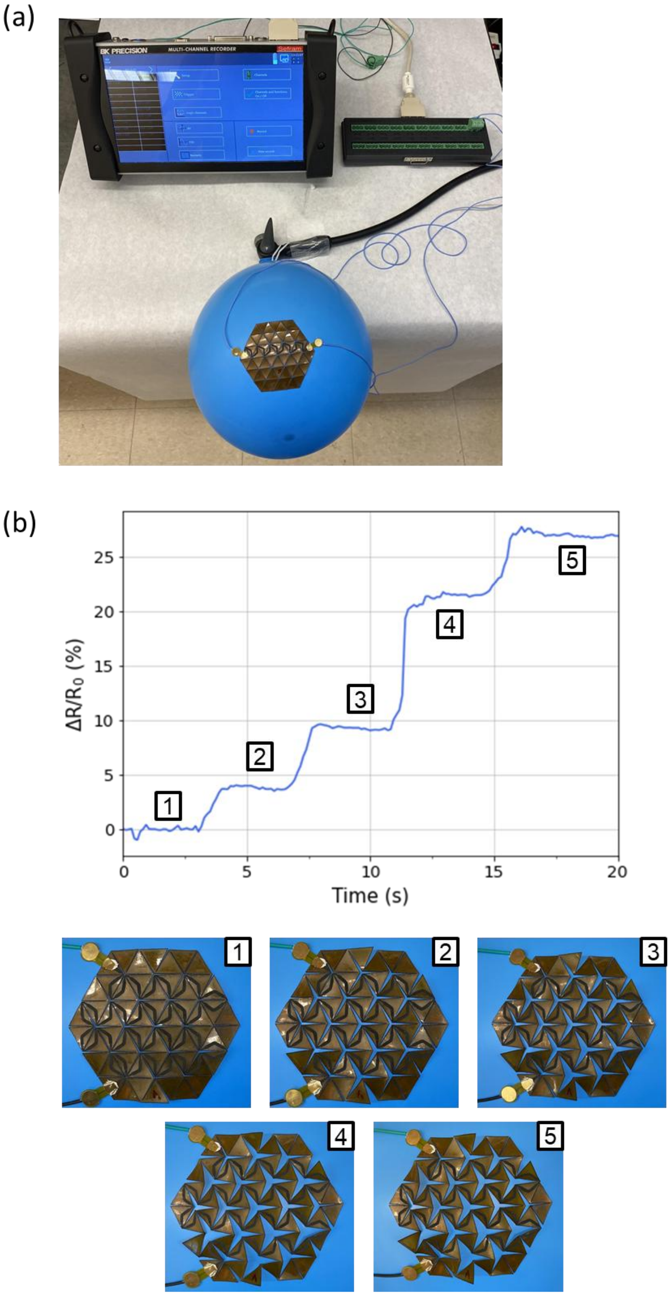

Figure S6. a) Experimental set-up for KLIG sensor attached to balloon and measurement of change in electrical resistance. **b)** Simulated bloating measurement using KLIG sensor with trace spanning whole surface attached to balloon surface using adhesive on the triangular faces. Pictures from 1-5 show the effects of ongoing inflation on the kirigami pattern.

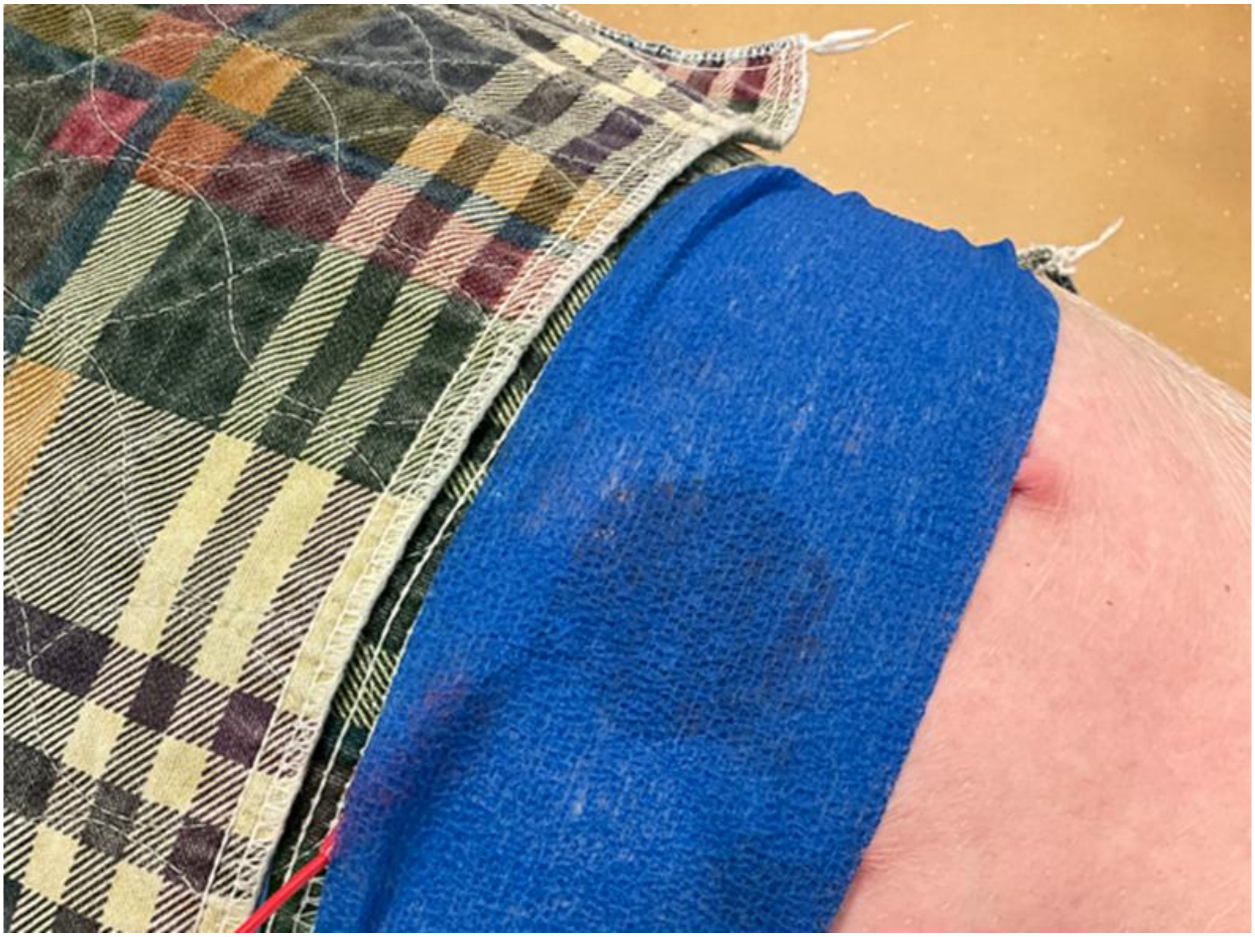

Figure S7. KLIG sensor attached to swine abdomen and affixed with veterinary wrap.

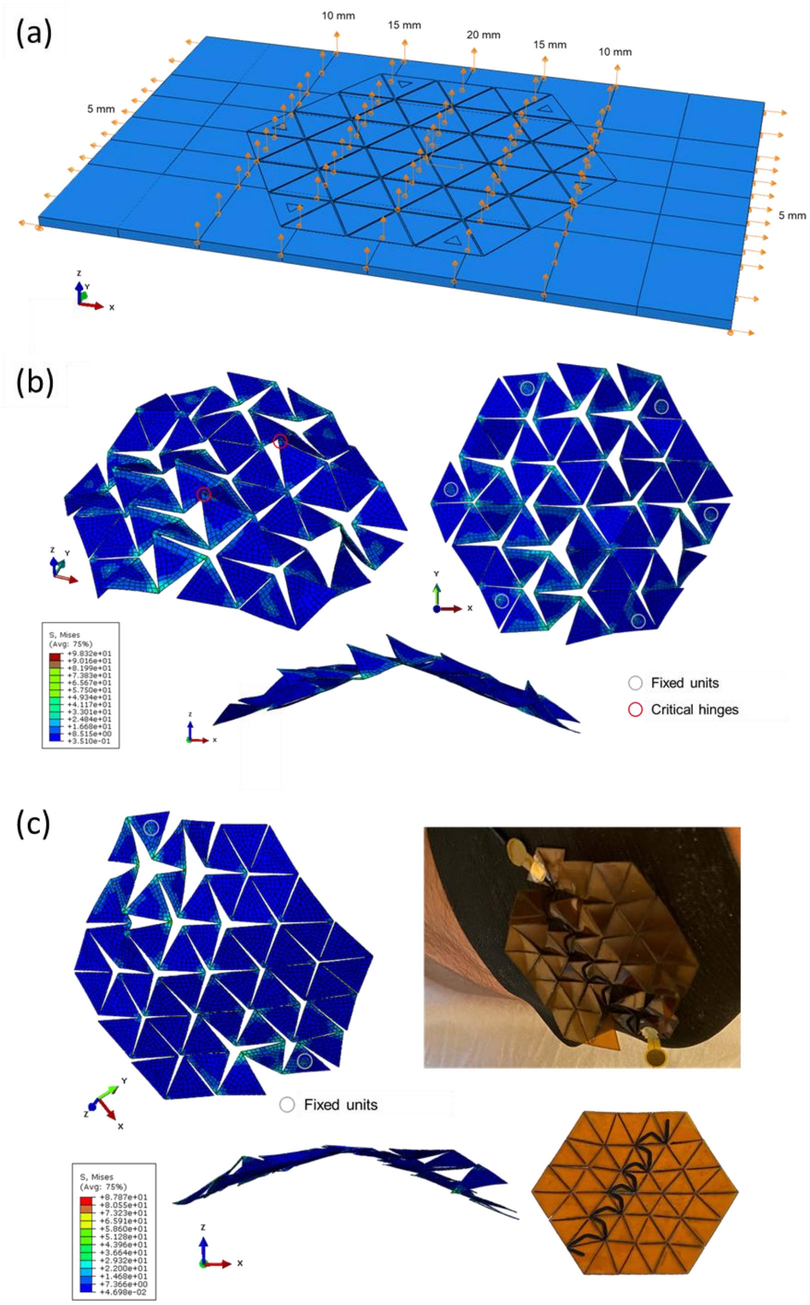

Figure S8. Validation of the base design modifications of the sensor when fixed to the elbow with FEA simulations. **a)** Boundary conditions set in Abaqus for simulating a stretching of the elbow. **b)** Simulation results for the elbow use case with the sensor being fixed at six outer units to the supporting material indicating major deformations in x- and z-direction. **c)** Modification of the sensor with decreasing unit size in the center and the trace being placed along the x-direction where the highest deformations take place. Only fixing the sensor at two corner minimizes the stress concentration at the critical hinges as in b).

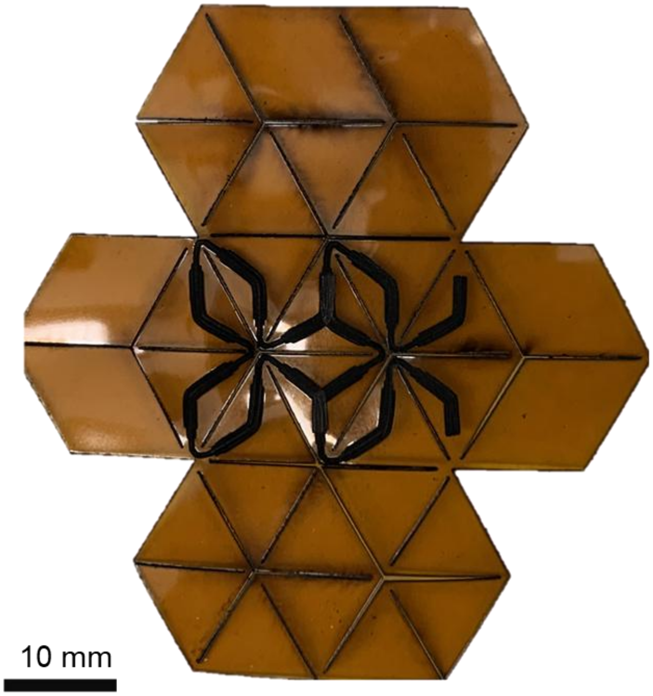

Figure S9. Modified KLIG sensor for pulse measurement of the pulse on the radial artery of a human volunteer. Triangular pattern was shifted to facilitate attachment.
